# Dynamic regulation of mitochondrial pyruvate metabolism is necessary for orthotopic pancreatic tumor growth

**DOI:** 10.1186/s40170-021-00275-4

**Published:** 2021-11-08

**Authors:** Nancy P. Echeverri Ruiz, Vijay Mohan, Jinghai Wu, Sabina Scott, McKenzie Kreamer, Martin Benej, Tereza Golias, Ioanna Papandreou, Nicholas C. Denko

**Affiliations:** 1 Department of Radiation Oncology, OSUCCC and Wexner Medical Center, The Ohio State University, Columbus, Ohio 43210 USA; 2 Current address: Hollings Cancer Center, Medical University of South Carolina, Charleston, SC 29425 USA; 3 Institute of Virology, Biomedical Research Center, Slovak Academy of Sciences, Bratislava, 84505 Slovak Republic

**Keywords:** Hypoxia, Glucose metabolism, Orthotopic pancreatic tumors, Pyruvate dehydrogenase, Mitochondria

## Abstract

**Background:**

Pyruvate dehydrogenase complex (PDC) plays a central role in carbohydrate metabolism, linking cytoplasmic glycolysis to the mitochondrial tricarboxylic acid (TCA) cycle. PDC is a conserved E1-E2-E3 dehydrogenase with a PDHA1 and PDHB heterotetramer functioning as the E1 subunit. PDHA1 contains three serine residues that can be reversibly phosphorylated by a dedicated family of four inhibitory pyruvate dehydrogenase kinases (PDHK1–4) and two reactivating phosphatases (PDP1, 2). Hypoxia induces the expression of PDHK1 and PDHK3 and hyperphosphorylates PDHA1. The role of PDC in metabolic reprogramming and tumor progression appears to be for the integration of oncogenic and environmental signals which supports tumor growth.

**Methods:**

To isolate the function of the serine-dependent regulation of PDC, we engineered MiaPaca2 cells to express PDHA1 protein with either intact serines at positions 232, 293, and 300 or all the combinations of non-phosphorylatable alanine substitution mutations. These lines were compared in vitro for biochemical response to hypoxia by western blot, metabolic activity by biochemical assay and Seahorse XF flux analysis, and growth in media with reduced exogenous metabolites. The lines were also tested for growth in vivo after orthotopic injection into the pancreata of immune-deficient mice.

**Results:**

In this family of cells with non-phosphorylatable PDHA1, we found reduced hypoxic phosphorylation of PDHA1, decreased PDH enzymatic activity in normoxia and hypoxia, decreased mitochondrial function by Seahorse flux assay, reduced in vitro growth of cells in media depleted of lipids, and reduced growth of tumors after orthotopic transplantation of cells into the pancreata of immune-deficient mice.

**Conclusions:**

We found that any substitution of alanine for serine at regulatory sites generated a hypomorphic PDC. However, the reduced PDC activity was insensitive to further reduction in hypoxia. These cells had a very modest reduction of growth in vitro, but failed to grow as tumors indicating that dynamic PDC adaptation to microenvironmental conditions is necessary to support pancreatic cancer growth in vivo.

## Background

The pyruvate dehydrogenase complex (PDC) is a large multi-subunit complex of molecular mass 9.5 MDa that is primarily located in the mitochondrial matrix where it catalyzes the irreversible decarboxylation and oxidation of pyruvate into acetyl-CoA, CO_2_, and NADH [1]. PDC exists as an evolutionarily conserved E1-E2-E3 dehydrogenase structure with the E1 subunit comprising a PDHA1_2_PDHB_2_ heterotetramer. Recent studies have linked altered PDC function to metabolic diseases, response to ischemic injury, and cancer [2–5]. PDC is integral to mitochondrial tricarboxylic acid cycle (TCA) function because it produces glucose-derived acetyl-CoA to be combined with oxaloacetate at citrate synthase to produce citrate. Mitochondrial citrate can either be incorporated in the TCA cycle or exported to the cytoplasm for the production of acetyl-CoA by ATP citrate lyase [6, 7]. This is a major source of cytoplasmic acetyl groups for fatty acid and cholesterol biosynthesis, as well as protein and histone modification [6, 7].

Regulation of PDC activity occurs at many levels. In addition to substrate level regulation, reports in the literature indicate that post-translational modifications such as tyrosine and serine phosphorylation, acetylation, and protein degradation can all alter PDC activity in response to different signals [8–10]. However, the most well-studied is PDC regulation by reversible serine phosphorylation of PDHA1 [11]. The dedicated family of pyruvate dehydrogenase kinases can add inhibitory phosphorylations to serine residues on PDHA1 at positions 232, 293, or 300 [12]. Modification of any one of the PDHA1 regulatory serine residues is sufficient to inactivate PDC catalytic activity [13]. These four PDH kinases are structurally similar to bacterial histidine kinases, supporting the model of a prokaryotic precursor of mitochondria [14].

Interestingly, PDC appears to be a “Goldilocks” enzyme complex that requires “just enough” activity. We, and several other groups, have shown that loss of inhibitory PDHK1 and hyper-activation of PDC is not compatible for growth of transplanted tumors in mice [15–17]. Other groups have shown that complete loss of PDC activity through inactivation of PDHA1 also stops the growth of tumors in some [18], but not all contexts [19].

**Fig. 1:**
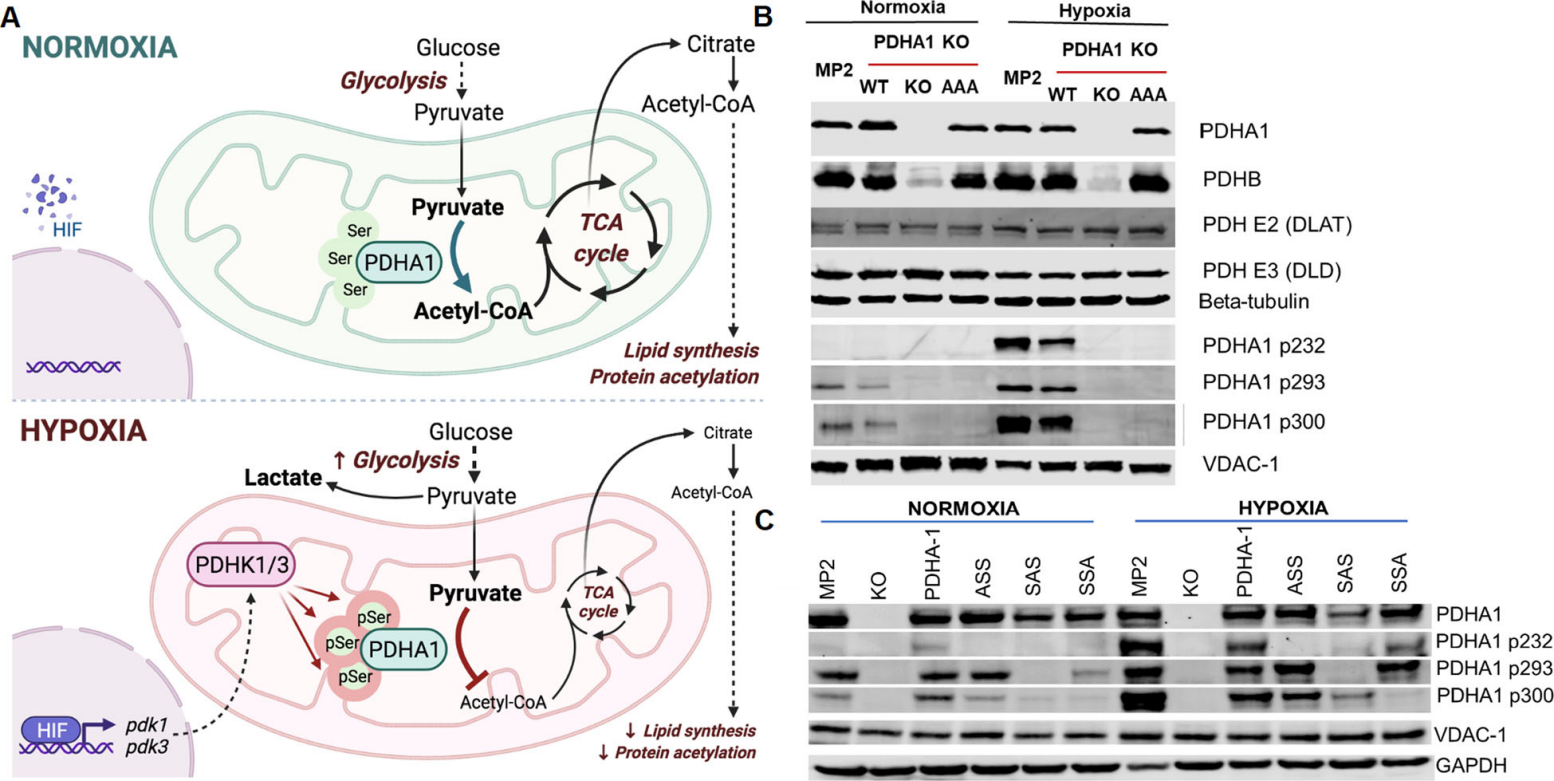
Effect of PDHA1 engineering on PDC components and PDHA1 phosphorylation during hypoxia. **A** Schematic showing the flux of glucose-derived pyruvate in normoxia (top) and hypoxia (bottom). Note that in hypoxia, HIF1-induced expression of PDHK1/PDHK3 leads to hyperphosphorylation of PDHA1 and attenuated flux through PDH to produce acetyl-CoA for citrate production at citrate synthase. **B** Western blot of lysates from parental MiaPaca2 and engineered cells with the indicated alleles after growth in complete media after 16 h at either 21% or 1% oxygen showing consistent levels of E2 and E3, near complete loss of PDHB, and no change in VDAC and similar levels of phosphorylation of regulatory serine residues in parent and engineered wild type line. Note VDAC as a mitochondrial control, and beta tubulin for loading control. One of *N*=3 replicates is shown. **C** Western blot of parental MiaPaca2 and engineered cells grown in complete media for 16 h at either 21% or 1% oxygen as indicated. NB there appears to be sequential phosphorylation from 293 to 300 to 232 as mutation in upstream sites affects downstream efficiency of modification. One of *N* = 3 replicates is shown. AAA, PDHA1 with alanine substitutions at serines 232, 293, and 300. AAS, PDHA1 with an alanine substitution at serine 300. ASA, PDHA1 with an alanine substitution at serine 293. SAA, PDHA1 with an alanine substitution at serine 232

Metabolic adaption of cancer cells in hypoxic microenvironments is largely regulated by the hypoxia-inducible transcription factor HIF-1. After hypoxic stabilization of HIF1α and translocation of HIF1α/HIF1β to the nucleus, HIF1 transactivates dozens of target genes, many of which are designed to reduce hypoxia [20]. PDHK1 [21] and PDHK3 [22] are direct HIF1 target genes that combine to reduce mitochondrial pyruvate oxidation and oxygen consumption (Fig. 1A). Together, they reduce oxygen demand in the tumor and work to bring oxygen supply and demand back into balance, reducing hypoxia [23].

In order to specifically investigate the role of inhibitory serine phosphorylation on PDHA1, we engineered the family of PDHA1 alanine point mutants at all the serine regulatory residues. After engineering cells to express only the mutant alleles, we found that point mutations in PDHA1 did not result in hyperactive PDC, but hypomorphic PDC. While this result was unexpected, the modified PDC was also insensitive to further inhibition by hypoxia. The cells with modified PDC also had reduced mitochondrial oxidation of pyruvate. Interestingly, this intermediate level of PDC activity was sufficient for growth in vitro, but not sufficient for orthotopic tumor growth. These findings indicate that hyperactive PDC (after PDHK1 KO) or complete loss of PDC activity (by PDHA1 KO) are both unable to support model tumor growth, indicating the importance of dynamic regulation of PDC.

## Materials and methods

### Cell lines and reagents

MiaPaca2 cell line was obtained from ATCC (American Type Culture Collection, USA). Cells were cultured in DMEM (Thermo Fisher) and carbon sources as indicated and FBS (10%) (Gibco) or charcoal-stripped FBS (10%) (HyClone). All were supplemented with 100 U/mL of penicillin and 100μg/mL streptomycin (Life Technologies). Cells were cultured at 37 °C in a standard cell incubator with humidified room air (5% CO_2_) or in a humidified Hypoxygen H35 workstation (1% O_2_, 5% CO_2_, 96% N_2_).

### Western blot and antibodies

Cells were lysed with RIPA buffer containing protease and phosphatase inhibitors (5 mM sodium fluoride, 2 mM β -glycerophosphate, 1 mM sodium orthovanadate, 1 mM phenylmethylsulfonyl fluoride (PMSF), and complete mini protease inhibitor cocktail (Roche)), 20–50 μg of total proteins electrophoresed and blotted to PVDF, and probed with anti: PDHA1 (1:6000, MitoSciences), pSer293-E1α (1:1000, EMD Chemicals), pSer300-E1α (1:1000, EMD Chemicals), pSer232-E1α (1:3000, EMD Chemicals), PDHK1 (1:4000, Assay Designs), PDHK2 (1:500, Novus), PDHK3 (1:1000, Novus), PDHK4 (1:1000, Novus), PDP1 (1:1000, Sigma Aldrich), HIF1α (1:1500, BD), and GAPDH (abcam). Primary antibodies were detected with fluorochrome-labeled secondary antibodies (Li-Cor) visualized on a Li-Cor Odyssey.

### Plasmids and transfection

Stable knockout cell lines were created using the Nickase cas9 system and a pair of guide RNAs. Cells were co-transfected with pTKhygro, and pX335-crisprPDHA1 A/pX335-crisprPDHA1 B or pX335-crispr PDHK1A/pX335-crisprPDHK1B using Lipofectamine 2000 (Invitrogen), followed by selection in 300 μg/mL hygromycin for 48 h and plated for single-cell cloning in drug-free media.

Colonies were tested by western blotting, and at least three positive clones were randomly pooled for study. The gRNA/CAS9 nickase plasmids included targeting sequences cloned into the Bbs1 site of pX335-U6-Chimeric-BB-CBh-hSpCas9n(D10A) (Addgene plasmid # 42335).

PDHA1a: 5′ GTGAGACCTCCCGGGCGGGC 3′

PDHA1b: 5′ GAGAAGCGCCAGACAGCACG 3′

PDHK1a: 5′ CCAGGGTGTGATTGAATACA 3′

PDHK1b: 5′ TGGGAATGACATCATTGTGT 3′

### Site-directed mutagenesis

hPDHA1 human untagged clone was obtained in a pCMV6-XL5, and it was subcloned into the Asc1 and Pme1 sites of pLenti-C-Myc-DDK-IRES-Puro Lentiviral Gene Expression Vector (addgene). Mutations were introduced using the QuikChange II Site-Directed Mutagenesis Kit (Agilent). Primers used for single/double and triple point mutations are as follows:

S232A forward 5′ GGAATGGGAACGGCTGTTGAGAGAGC3′, reverse 5′ GCTCTCTCAACAGCCGTTCCCATTCC 3′

S293A forward: 5′ GTTACCACGGACACGCTATGAGTGACCCTG 3′, reverse: 5′ CAGGGTCACTCATAGCGTGTCCGTGGTAAC 3′

S300A forward: 5′ GTGACCCTGGAGTCGCTTACCGTACACGAG 3′, reverse: 5′ CTCGTGTACGGTAAGCGACTCCAGGGTCAC3′

Plasmid clones were confirmed by Sanger sequencing at the Genomics Shared Resource (GSR) at The Ohio State University CCC (OSUCCC).

### PDC immunocapture activity assay

PDH activity was measured using the pyruvate dehydrogenase (PDH) Combo (Activity + Profiling) Microplate Assay Kit (ab110671); 10–20 × 10^6^ cells were pelleted and resuspended in 400 μL PBS; 50 μL of resuspended cells were lysed to measure the protein concentration (protein should be 15 mg/mL). Fifty microliters of detergent solution was added to the 450 μL of cell suspension and incubated on ice for 10 min. The suspension was centrifuged at 1000×*g* for 10 min at 4 °C, and the supernatant was collected and transferred to a clean tube on ice. Samples are diluted to the desired concentration in 1× sample buffer. Antibody-coated 96-well plates are loaded with 200 μL of sample and 200 μL sample buffer and incubating for 3 h at room temperature. The sample is removed by inversion and wells washed 2× with 300 μL stabilizing solution. Two hundred microliters of assay solution is added to each well and immediately read every minute for 2 h in a plate reader at 450 nm at room temperature. For excess TTP and MgCl_2_ experiments, reagents were added to the assay buffer immediately before plate measurement. Enzyme activity was normalized to capture the amount of PDHA1 as indicated by western blot of protein removed from the assay well.

### Seahorse XF flux assay

1.5 × 10^4^ cells per well were plated in complete media with serum, and the next day, the media were changed to basal DMEM without serum containing only glucose as a carbon source. Two hours later, equilibrated plates were placed in the Seahorse device, and OCR was measured for 4–6 cycles to establish a baseline. Test compounds were added as indicated, and 4–6 cycles were run to establish their effects on OCR. Finally, 0.3 μg/mL antimycin A was added, and 4–6 more cycles of OCR were measured to distinguish mitochondrial OCR from total cellular OCR. Cell lines were tested in sextuple with three biological replicates.

### Clonogenic assay

Three hundred cells were plated in 6-cm dish; the next day, the media were changed as indicated, and colonies were left to form for 16 days in either normoxia or hypoxic conditions. Colonies were fixed with acetic acid: methanol (1:7), then stained with crystal violet and quantified using AlphaImager: Alpha Innotech Corporation. Colonies > 50 cells were scored.

### Orthotopic xenografts

10^6^ MiaPaca2 PDAC cells or the indicated derivates were implanted in immune-deficient nude mice following IACUC-approved protocols. Briefly, an incision was placed in the right flank of anesthetized animals the spleen and pancreas liberated. Cells were mixed 1:1 with Matrigel and implanted in the tail of the pancreas in 10 μL. Incision was sutured in 2 layers, and animals were given long-lasting analgesics and allowed to recover from anesthesia. Mice were returned to housing for post-op care and monitored daily for 4 days. Animals were checked weekly by palpation to determine tumor growth.

### Statistical analysis

Statistical analyses were performed using either Microsoft Excel or Graphpad Prism. Pair comparisons were made using Student’s *T* test, and multiple comparisons using ANOVA. Statistical significance was determined if *P*< 0.05.

## Results

### Generation and characterization of cells expressing PDHA1 with engineered alanine mutations at regulatory serine residues 232, 293, and 300

To engineer cells that exclusively express mutant forms of PDHA1, we first knocked out an expression of the endogenous wild-type gene using a modified CRISPR/Cas9. We used the “Nickase” Cas9 that only cuts one strand of DNA, and therefore requires guide RNAs for both strands [24]. After transfection with plasmids expressing the nickase, both guide RNAs and pTKhygro, cells were selected with hygromycin for 2 days and plated for colony formation. Individual clones were screened by western blot for PDHA1 expression and groups of 6 null clones were pooled into 2 separate pools for the PDHA1 null MiaPaca2 lines. We then transfected these PDHA1 null cells with a PDHA1-IRES-puro^r^ plasmid containing either the WT PDHA1, or PDHA1 mutants that had been generated using a point mutagenesis strategy (Agilent Quick Change). These cells were selected for puromycin resistance, and PDHA1 expression was confirmed by western blot. These cells were used in the following experiments.

Figure 1B shows a western blot analysis of lysates from engineered MiaPaca2 cells to show how alteration of PDHA1 affected the levels of the other members of the PDC or any change in the overall level of mitochondria as measured by the level of the voltage-dependent anion channel (VDAC) protein. Examining the parental cells, PDHA1 KO and PDHA1 KO with either WT or triple alanine mutant S232A/S293A/S300A (AAA), we find that the levels of the introduced PDHA1s are similar to that of the endogenous gene (Fig. 1B). The E2 (dihydrolipoamide S-acetyltransferase (DLAT)) and E3 (dihydrolipoamide dehydrogenase (DLD)) components of the PDC are also unchanged in the engineered lines. However, the direct binding partner of PDHA1, PDHB, that combines to form the E1 subunit as an A_2_B_2_ heterotetramer is almost entirely absent in the PDHA1 KO line, indicating that without its binding partner, PDHB appears to be unstable. Using phospho-specific antibodies, we also find comparable levels of hypoxic phosphorylation of the three regulatory serine residues in the parental cells and the cells with WT PDHA1 reintroduced, but no phosphorylation in the cells with the AAA mutant reintroduced. Finally, we detected no significant difference in the level of the mitochondrial protein VDAC, indicating no dramatic change in the total amount of mitochondria (Fig. 1B).

We next examined the effects of the single point mutants on the hypoxic induction of phospho-PDHA1 using phospho-specific antibodies. Figure 1C shows what appears to be a sequential series of phosphorylations. We find that alanine at position 293 significantly reduces phosphorylation at both sites 300 and 232. Likewise, alanine at 300 did not appear to have a significant impact on 293 phosphorylation but does decrease 232 phosphorylation. Finally, alanine at 232 did not appear to have a significant impact on phosphorylation at either serine 293 or 300. We interpret this to show a sequential series of phosphorylation events from 293 to 300 to 232, perhaps by the same kinase molecule. When one phosphorylation event is blocked by mutation, the downstream events are significantly reduced. This is consistent with what has been reported with phosphorylation events in isolated rat heart PDH [25].

**Fig. 2:**
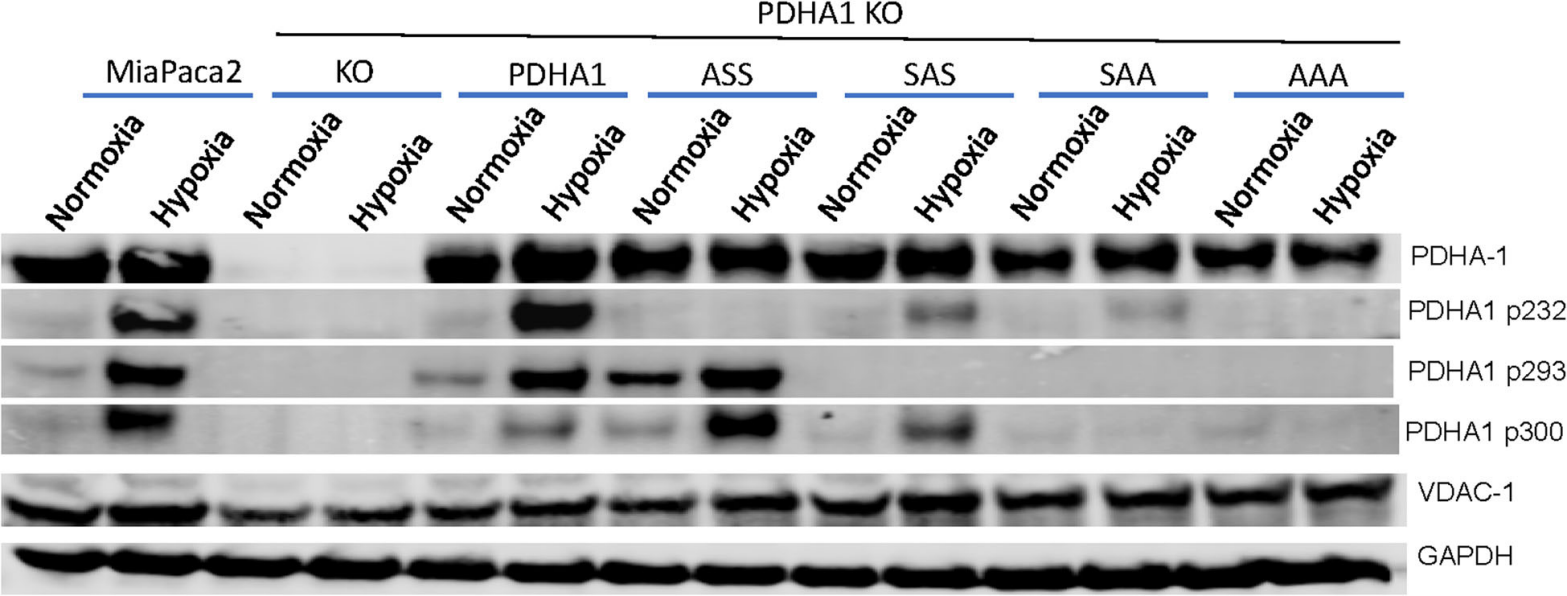
Hypoxic response of compound mutant alleles of PDHA1. Western blot analysis of lysates from indicated engineered cell lines after 16 h in 21% or 1% oxygen. PDHA1 serine 232 phosphorylation is significantly reduced by single mutations of either 293 or 300, with almost a complete block when both are mutated. N.B. Mutation of all three sites completely abrogates hypoxic PDHA1 phosphorylation as detected by immunoreactivity. One of *N* = 3 replicates is shown. ASS, PDHA1 with alanine substitutions at serines 293 and 300. SAA, PDHA1 with alanine substitutions at serines 293 and 300

We were interested in the unique nature of serine 232 because it can only be phosphorylated by PDHK1, while the other residues can be phosphorylated by all the PDHKs [13]. We therefore examined the mutant alleles that only had a serine at 232, S293A/S300A (SAA), and the allele that only had an alanine at the serine at 232 S232A (ASS). Examination of cells expressing these alleles supports the model that 232 is the last residue to be phosphorylated. Figure 2 shows that mutation of 232 has very little effect on hypoxic phosphorylation of 293 and 300, while mutation of 293 and 300 reduces the amount of phosphorylation of 232 to almost undetectable levels (Fig. 2).

### Phosphorylation site mutations in PDHA1 reduce PDC enzymatic activity

**Fig. 3:**
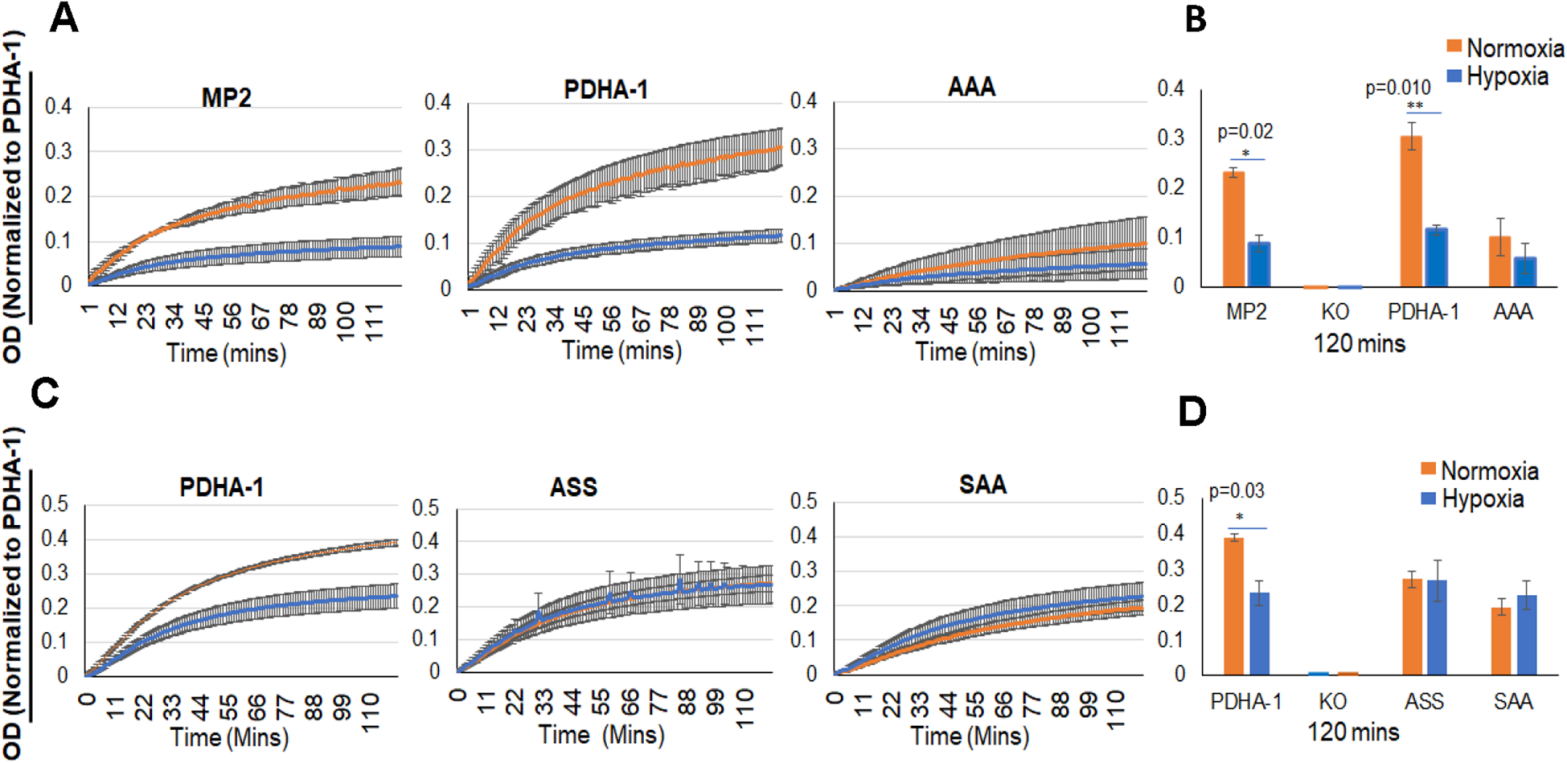
PDHA phospho-site mutants decrease enzymatic activity of PDC. Pyruvate dehydrogenase enzymatic activity microplate immunocapture assay (abcam ab110671) was used to quantify the PDC activity in MiaPaca2 and phospho-site mutant cells after culture for 16 h in 21% or 1% oxygen. Each sample was normalized to captured PDHA1 as determined by western blot of captured protein. **A** Kinetic readings of parental MiaPaca2, wild-type PDHA1 re-expressed, and S-232,293,300-A (AAA) PDHA1 re-expressed. Results are the average of 3 experiments, and PDHA KO cells had 0.00 absorbance (data not shown). *N* = 3 biological replicates. **B** Endpoint measures for curves reported in **A** with statistical significance calculated by *T* test. **C** Focus on the significance of serine 232 with analysis of PDHA1 mutants S232A (ASS) and S293A/S300A (SAA). These two mutants show similar activity to the S232A/S293A/S300A mutant assayed A and B. However, the enzymes are still insensitive to hypoxia. *N* = 3, *P*=NS. **D** Endpoint measures for curves reported in **C** with statistical significance calculated by *T* test. Error bars, s.e.m. *P< 0.05, **P< 0.01

We hypothesized that by removing the inhibitory serine phosphorylation residues on PDHA1, we would generate a constitutively active PDC that would not be inhibited in hypoxia. We therefore biochemically measured PDC activity after immunocapture from cells grown in normoxia or hypoxia. The commercial assay (PDH Activity Microplate Assay, Abcam ab110671) used the generation of NADH and conversion of a chromogenic substrate from the captured PDC. Counterintuitively, we found a significant reduction in enzymatic activity in cells expressing the AAA mutant PDHA1 (Fig. 3A, B). Interestingly, the reduced activity in the AAA PDC is not further reduced after treatment of cells with hypoxia. We next tested mutants that would inform about the significance of the 232 residue that is unique in its phosphorylation by PDHK1. We performed a PDC activity assay from normoxic and hypoxic cells expressing S232A (ASS), and S293A/S300A (SAA) for activity. Figure 3C and D show that PDC containing either of these mutants also have reduced activity that is resistant to hypoxia.

**Fig. 4:**
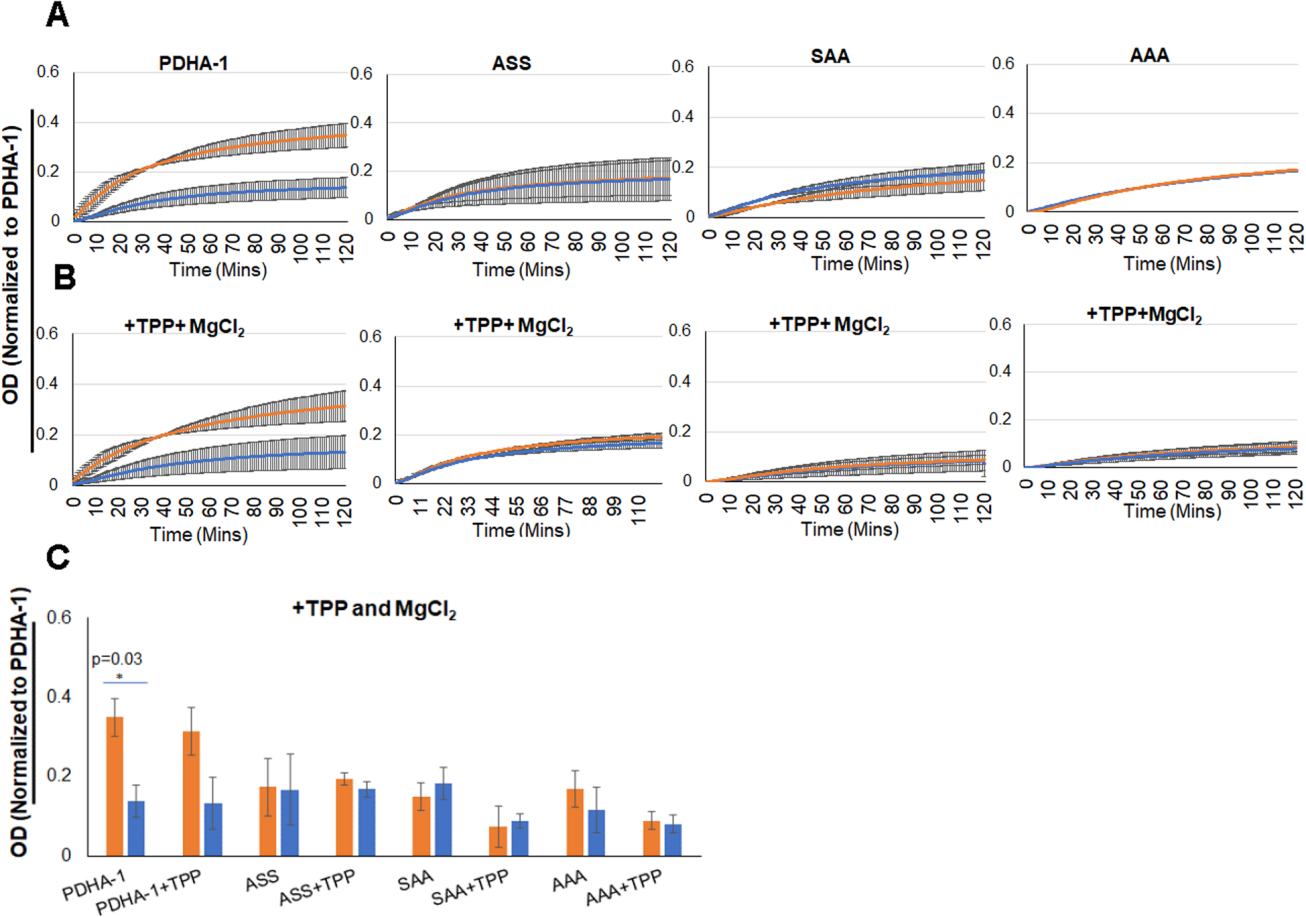
Addition of 10× TPP and MgCl_2_ does not rescue enzymatic activity of PDC in PDHA1 mutants. PDC microplate activity assay following immunocapture in standard assay buffer, or assay buffer supplemented with 10× increased TPP (200μM) and MgCl_2_ (10 mM). Each sample was normalized to the captured PDHA1 as measured by western blot of captured proteins. Cells were treated for 16 h in either 21% or 1% oxygen as indicated. **A** Standard assay buffer. Kinetic assay results of wilt type and mutant expressing cell lines as indicated. **B** Reaction buffer with excess thiamine pyrophosphate and magnesium chloride. Kinetic results of wild type and mutant expressing cell lines as indicated. **C** Endpoint measure from results reported in A and B. Error bars, s.e.m. *P< 0.05

One possible explanation for the reduced activity of the mutant enzymes would be decreased affinity for the essential co-factor thiamine pyrophosphate (TPP). The binding site for TPP as well as pyruvate is related on the surface of PDHA and includes the area around the regulatory serine residues [26]. We therefore repeated the PDC activity assay, but after immunocapture, we increased the TPP and MgCl_2_ concentrations tenfold in the assay buffer. We find that increasing cofactors did not rescue the enzymatic activity, indicating that low affinity for TPP was probably not the cause for reduced PDC activity (Fig. 4).

### Decreased PDC activity causes decreased mitochondrial oxygen consumption

**Fig. 5:**
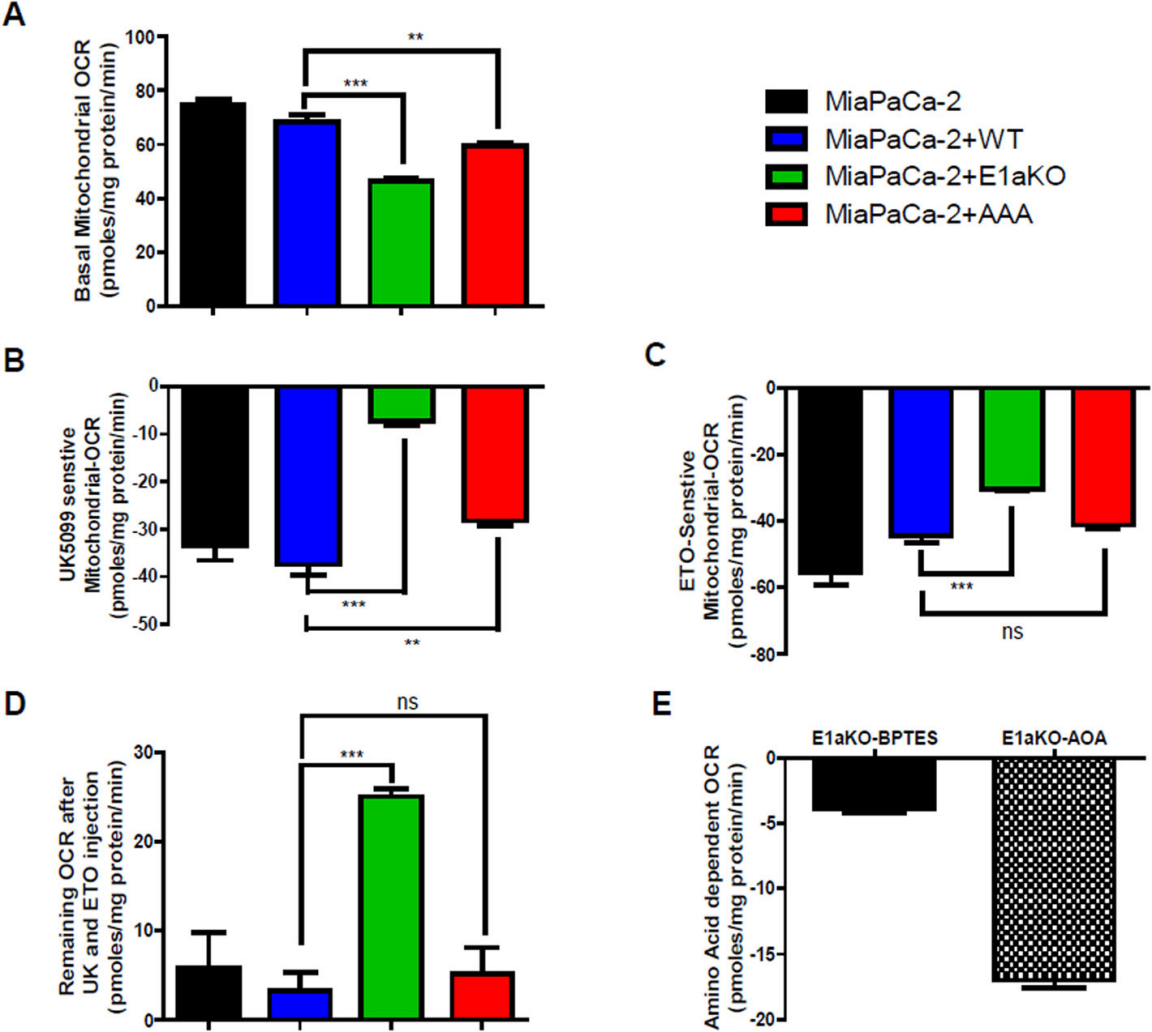
Mitochondrial function in cells with engineered PDHA1. **A** Basal mitochondrial (sensitive to 0.3 μM antimycin) OCR of the indicated cells grown in media with only 5 mM glucose as a carbon source. *N* = 3 biological replicates *P< 0.05, **P< 0.01, ***P< 0.001. **B** UK5099 (10 μM) sensitive OCR in the same cells and media as in **A**. *N* = 3. **C** Etomixir (68 μM) sensitive OCR in the same cells and media as in **A**. *N* = 3. **D** Remaining mitochondrial OCR after both UK5099 and Etomixir OCR in the same cells and media as in **A**. *N* = 3. **E** PDHA1 KO cell mitochondrial OCR sensitivity after treatment with either glutaminase inhibitor BPTES (8 μM) or transamination inhibitor AOA (1 mM) in the same media as **A**. *N* = 3

We next investigated the impact of engineered PDH variants on overall mitochondrial function. PDC is a major entry point for glucose-derived carbons to fuel the TCA cycle, so we hypothesized that decreased PDC activity would have a significant impact on mitochondrial oxygen consumption, especially when cells were in media containing only glucose as a metabolic fuel. We performed Seahorse XF analysis on cells that had been equilibrated for 2 h in basal media without serum, and with only 5 mM glucose as a carbon source. Under these conditions, we find that complete deletion of PDHA1 resulted in a 33% decrease in mitochondrial OCR as measured by Seahorse XF flux analysis (PDHA1 KO 46 pM O_2_/min/mg versus PDHA1 KO with WT PDHA1 reintroduced at 69 pM/min/mg) (Fig. 5A). We also find that cells expressing the hypomorphic allele PDHA1 AAA mutant have an OCR that is intermediate between that in the WT PDHA1 and PDHA1 KO cells (59 pM/min/mg, *P*=0.01 versus WT).

In order to determine how these mitochondria adapted to alterations in PDC, we further analyzed the mitochondrial metabolism using inhibitors of mitochondrial pyruvate carrier (10 μM UK5099), fatty acid oxidation by Cpt inhibitor (68 μM etomoxir), and glutamine oxidation by glutaminase inhibitor (8 μM BPTES). Panel 5B shows that the UK5099-sensitive OCR is 37 pM/min/mg protein for the KO cells reconstituted with WT PDHA1, 28 if reconstituted with AAA PDHA1, and only 7 for the PDHA1 null cells. Panel 5C shows the etomoxir sensitive OCR in the same cells and find that WT and AAA expressing cells have an equivalent reduction in OCR (44 pM/min/mg for the WT, 41 for the AAA mutant) but there is a reduced decrease in OCR for the PDHA1 KO (30 pM/min/mg). All cells showed a similar minimal BPTES sensitive OCR (< 6 mM). Subtracting UK5099 sensitive- and ETO sensitive- from total OCR shows that there was additional unaccounted for OCR substrate in the PDHA1 KO resulting in over 25 pM O_2_/min/mg. To investigate what fuel source this might be due to, we tested the PDHA1 KO cells for oxidation of amino acids by measuring sensitivity to the glutaminase inhibitor BPTES and the transamination inhibitor aminooxyacetate (AOA). We found that 17 pM/min/mg of the PDHA1 KO OCR was indeed sensitive to AOA, indicating the PDHA1 KO cells adapt by using an amino acid(s) such as valine, isoleucine, or leucine as this alternative mitochondrial fuel (Fig. 5E).

### PDHA1 loss increases demand for uptake of exogenous lipids

**Fig. 6:**
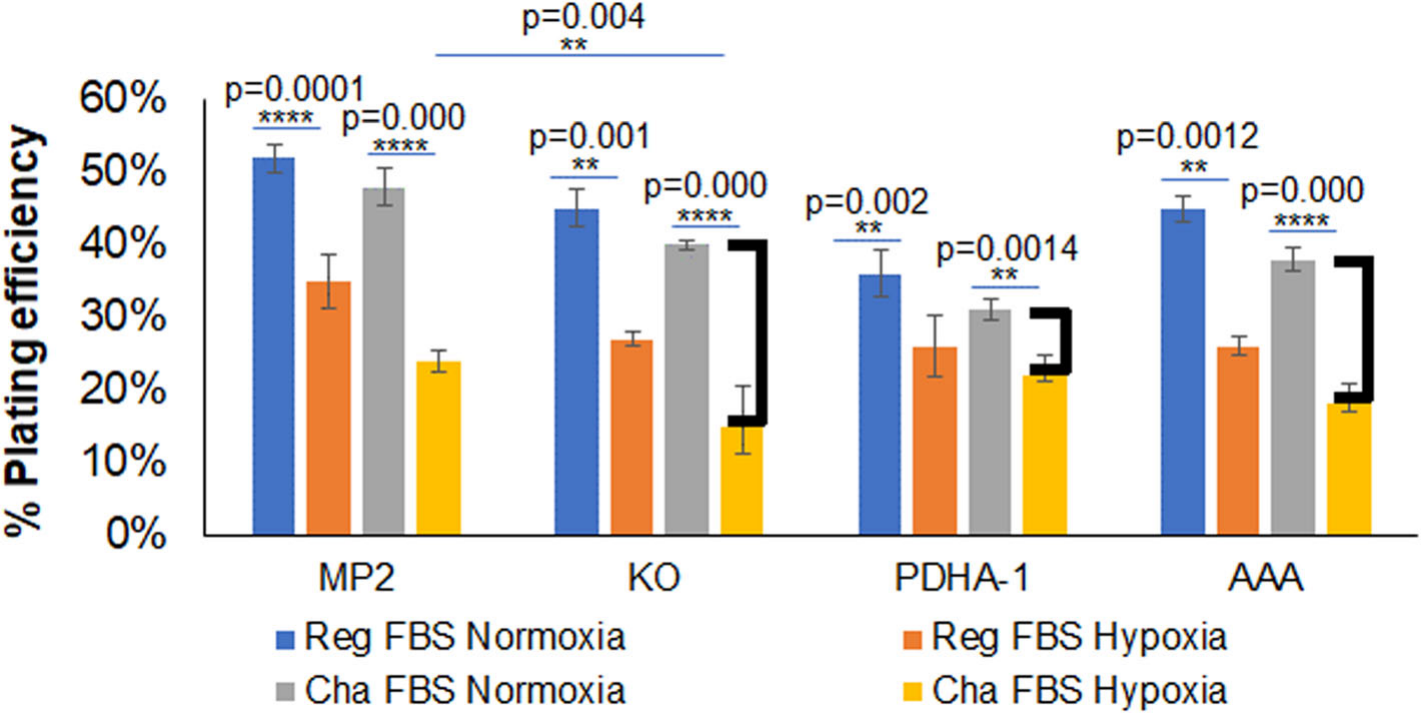
PDHA1 supports the growth of tumor cells in media without exogenous lipids. Cell colony formation was measured in DMEM media containing either 10% complete or 10% charcoal-stripped FBS to remove non-polar compounds, in either 21% or 1% oxygen environment. Note decrease in plating efficiency in hypoxic, lipid-depleted media indicated. *N* = 3 biological replicates. Error bars, s.e.m. *P< 0.05, **P< 0.01, ***P< 0.0001, ****P = 0

PDC is an important component of de novo lipogenesis because PDC produces acetyl-CoA for citrate production at citrate synthase. The mitochondrial pool of citrate fuels the TCA cycle, but it is also shuttled to the cytoplasm for conversion back to acetyl CoA at ATP citrate lyase [7]. This is a major source of acetyl-CoA for the production of fatty acid and cholesterol. We therefore tested the engineered cells for their ability to grow in culture without exogenous lipids. Cells were plated for colony formation in complete media with either 10% standard FBS, or complete media with 10% charcoal-stripped FBS that has had non-polar compounds removed. Half the dishes were placed in a 21% oxygen incubator, and half were grown in 1% oxygen environment for 12 days to allow colonies to form. Figure 6 shows that all lines appear to have a lower absolute plating efficiency after hypoxia, but the PDHA1 KO cells have the most significant hypoxic decrease in the plating efficiency when comparing colony formation in lipid depleted media in normoxia and hypoxia. The PDHA1 knockout decreases to 38% of its normoxic plating efficiency while the PDHA1 reintroduced cells decrease to 67% of their normoxic plating efficiency and the AAA mutant reintroduced cells to 52% of their normoxic plating efficiency. These results indicate that loss of PDHA1 sensitizes the growth of tumor cells to the removal of exogenous lipids when grown in hypoxia. The partial activity of the AAA mutant produces intermediate growth under these lipid-depleted and hypoxic conditions.

### PDHA1 and PDHK1 are necessary for the growth of pancreatic tumors

**Fig. 7:**
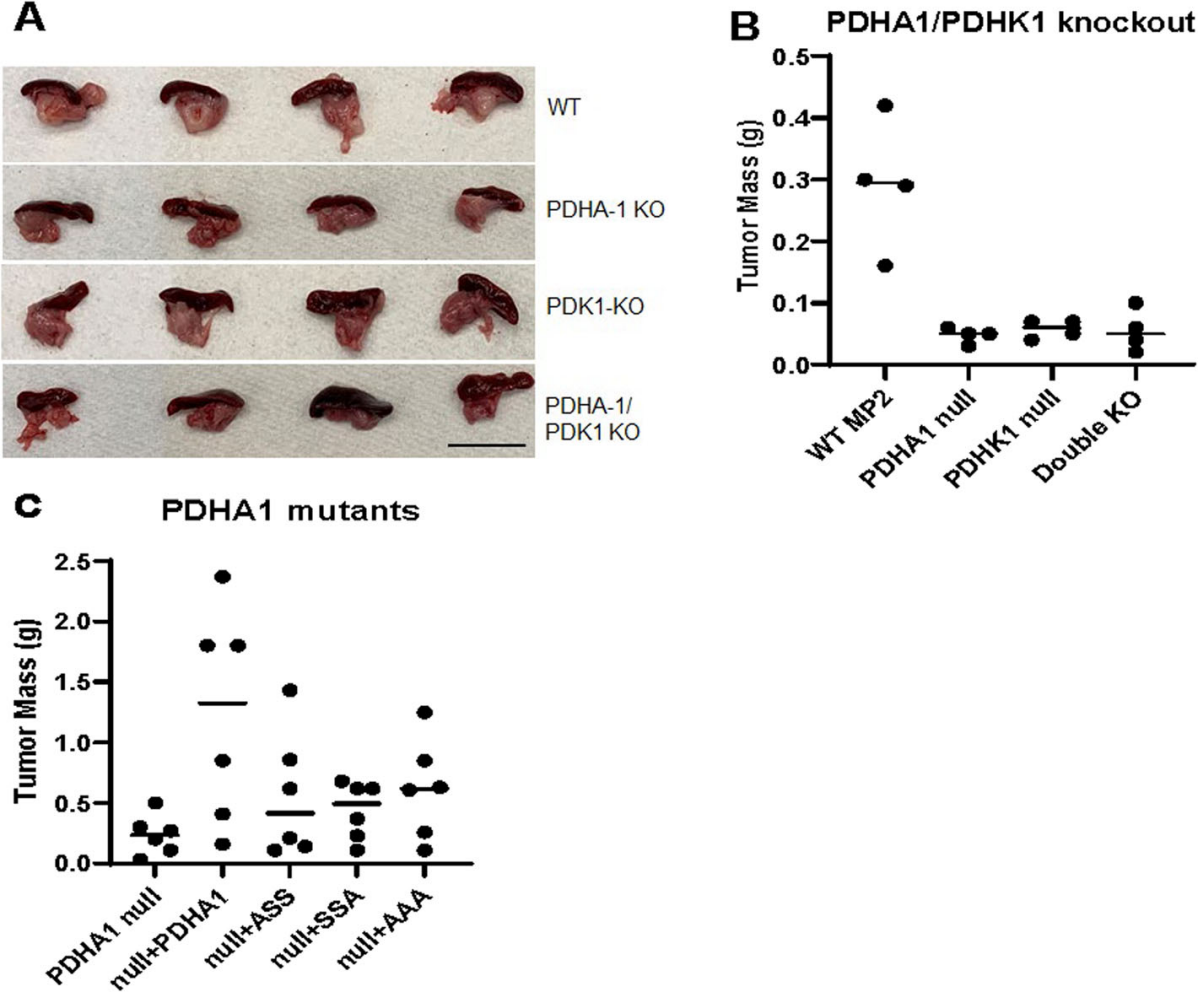
PDHA1 regulation and PDHK1 are essential for optimal growth of orthotopic pancreatic tumors. 10^6^ of the indicated cells were injected into the pancreas of immune-deficient mice. Tumor mass was calculated by taking the mass of the pancreas and spleen and subtracting 0.1 g for normal tissue. **A** Image of excised orthotopic tumors in the pancreas (inferior) and spleen (superior). **B** Individual pancreas masses from the indicated knockout MiaPaca2 cells 4 weeks after implantation. *P* = 0.003 by ANOVA. **C** Individual pancreas masses from tumors grown from PDHA1 mutant expressing MiaPaca2 cells 8 weeks after implantation as calculated in **B**. *P* = 0.029 by ANOVA

Tumor cells must reprogram their metabolic pathways to survive the microenvironmental conditions in the tumor where oxygen, glucose, and other nutrients can be limiting. We therefore tested the growth requirement for PDHA1 and PDHK1 in vivo. Metabolite availability has been shown to differ in model tumors grown in heterotopic versus orthotopic sites [27]. We therefore decided to implant WT, PDHA-1 KO, PDHK1 KO, and double PDHA-1/PDHK1 KO MiaPaca2 cells in the pancreas of immuno-compromised athymic nude mice. After 4 weeks, tumors were harvested (Fig. 7A). Measuring excised pancreas weight showed that only mice injected with WT cells developed measurable tumors (Fig. 7B), neither PDHK1 KO, PDHA1 KO, or double KO developed detectable tumors. These results are consistent with, and extend our previous findings indicating an essential role for PDHK1 regulation in the growth of heterotopic model pancreatic tumors [16]. These results also indicate that either too little PDC activity (PDHA1 KO) or too much PDC activity (PDHK1 KO**)** is not compatible with tumor growth.

We next tested the growth of tumor cells containing the hypomorphic alleles of PDHA1. The lines PDHA1 KO, PDHA1 KO reconstituted with WT PDHA1, PDHA1 reconstituted with either PDHA1 SAA, PDHA1 ASS, or PDHA1 AAA were injected into the pancreata of nude mice using the protocol described above, but tumor growth was extended to 8 weeks to allow for partial growth of slow-growing variants. Using an extended time of growth, we do see some growth of the PDHA1 knockout tumors (median weight of 0.21 g versus 0.05 g in Fig. 7A). The cells reconstituted with the wild-type enzyme grew to an average of 1.2 g, while the ASS, SAA, and AAA mutants all grew to approximately 0.5 g. These results indicate that a small amount of PDC activity from the hypomorphic enzymes did increase tumor growth, but well-regulated wild-type PDC activity is necessary for optimal growth of these model pancreatic tumors.

## Discussion

The pyruvate dehydrogenase complex (PDC) is an evolutionarily conserved enzyme that regulates much of the flux of pyruvate into the TCA cycle. The regulated flow of carbohydrate-derived carbons into the mitochondrial pathways appears to be essential for the growth of mammals. Germline mutations in human PDHA1 can cause neurological deficits, hypotonia, brain abnormalities, and lactic acidosis that is often fatal to infants [28]. Targeted deletion of Pdha1 in mice is embryonic lethal at approximately day 9.5 [29]. Evolutionarily, the number of inhibitory pyruvate dehydrogenase kinases increases from 1 in yeast S. cerevisiae to 4 in mammals. The need for this seemingly redundant number of independent regulatory kinase genes has not been clearly defined in the literature. Interestingly, this importance for the environmental regulation of PDC is even more apparent when we find that both PDHK1 and PDHK3 are hypoxia inducible [21, 22].

In vitro experiments indicate that phosphorylation of any one of the three serine residues on PDC is sufficient to inhibit enzymatic activity [13]. Interestingly, the serine at position 232 is uniquely phosphorylated by PDHK1 [12], but no functional significance has been placed on this biochemical relationship. Here, we find that 232 is the final serine to be phosphorylated, and without the other two serine residues on 293 and 300, phosphorylation of 232 is highly compromised (Fig. 2). This complicated relationship may be in part because PDHKs can function as homo and heterodimers [30]. Efficient binding of the kinases to PDC and PDHA1 phosphorylation may require heterodimerization, and this may facilitate PDHK1 activity on serine 232 on wild type PDHA1. Unfortunately, due to the unexpected loss of PDC activity after mutation of any of the serine residues, the functional significance of PDHA1 mutant SAA could not be determined in these studies.

Mitochondrial PDC is important for redox as well as carbon regulation [27]. PDC generates NADH as well as CO_2_ and acetyl-CoA supporting TCA reactions. The electron in NADH is passed to complex 1 of the electron transport chain. Cells require mitochondria as an electron sink to support biosynthetic processes, especially in hypoxia [31, 32]. Without mitochondrial ETC, alternative cellular sinks for electrons are required such as lactate dehydrogenase that converts pyruvate to lactate consuming NADH. However, several other important mitochondrial redox reactions contribute to NAD(P)H homeostasis such as serine hydroxymethyltransferase 2 (SHMT2).

PDC-generated acetyl-CoA is used to produce citrate at citrate synthase. Mitochondrial citrate can be translocated to the cytoplasm to be cleaved by ATP citrate lyase to generate acetyl-CoA and oxaloacetate [33]. This is a major source for cytoplasmic acetyl-CoA which is an important building block for macromolecular synthesis and a potential regulatory modification of other proteins. We have detected a modest in vitro dependence on exogenous lipids in the cells with PDHA1 KO, but this does not appear to explain the essential role in model tumor growth. Perhaps the dynamic roles of PDC on NAD(P)H regulation or histone/protein acetylation is more important for the growth of tumor cells in vivo.

## Conclusions

These findings indicate that the pyruvate dehydrogenase complex is an integral component of pancreatic cancer cell metabolism. PDC activity also needs to be regulated to provide optimal support of tumor cell growth in vivo. Regulatory phosphorylation of PDC is a dynamic process that appears to respond to changes in the tumor microenvironment to fine-tune pyruvate flux into the mitochondrial TCA reactions. Furthermore, these regulatory serine residues in PDHA1 are structurally important for enzyme activity. The modest change we engineered to a less polar alanine residue significantly slows the enzyme, possibly by altering substrate binding, but not by inhibiting co-factor binding. Careful structural analysis would be needed to determine if these changes have a significant impact on the E1 subunit of PDC, or on the formation of the multi-subunit PDC complex. Mutation of any of the 3 regulatory serine residues impinges on phosphorylation of the PDHK1-specific target S232, possibly due to subtle changes in surface charge or processivity of individual kinase molecules. In mice, the PDHK1 gene is dispensable, making PDHK1 an attractive target for anti-cancer drug development.

## Data Availability

All data generated in these studies are presented in the manuscript or supplementary associated data.

## Abbreviations

PDH: Pyruvate dehydrogenase
PDC: Pyruvate dehydrogenase complex
PDHK: Pyruvate dehydrogenase kinase
PDP: Pyruvate dehydrogenase phosphatase
ETC: Electron transport chain
TCA: Tricarboxylic acid cycle
HIF: Hypoxia-inducible factor
KO: Knockout
NADH: Nicotinamide adenine dinucleotide
OCR: Oxygen consumption rate
ECAR: Extracellular acidification rate
AAA: PDHA1 with alanine substitutions at serines 232, 293, and 300
AAS: PDHA1 with alanine substitutions at serine 300
ASA: PDHA1 with alanine substitutions at serine 293
SAA: PDHA1 with alanine substitutions at serine 232
